# Supplementary material for: Spatial-ID: a cell typing method for spatially resolved transcriptomics via transfer learning and spatial embedding
Source: Nat Commun. 2022 Dec 10;13:7640. doi: 10.1038/s41467-022-35288-0 (PMC9741613; doi:10.1038/s41467-022-35288-0)
Supplement: Supplementary file 1 — Supplementary imformation [file 41467_2022_35288_MOESM1_ESM.pdf]

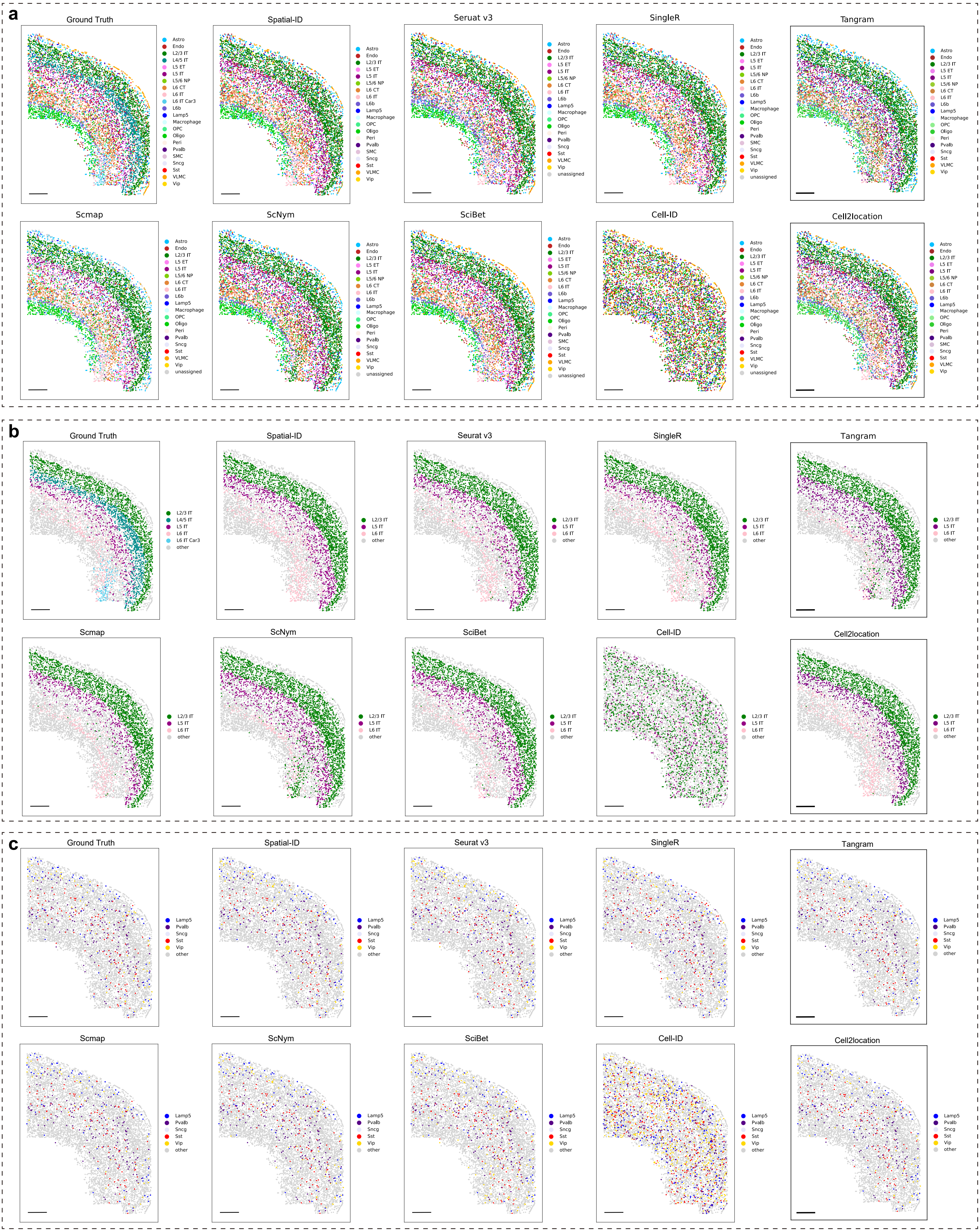

Supplementary Figure 1 | Spatial organization of ground truth and predictions of different cell typing methods. Bar scale 400um. (**a**) All cell types. (**b**) Intra-telencephalic neurons (L2/3 IT, L4/5 IT, L5 IT, L6 IT and L6 IT Car3). Notably, L4/5 IT and L6 IT Car3 neurons are not provided in reference dataset, thus the predictions of all cell typing methods do not recall them. As shown in Fig. 2k, the extended postprocess (new cell type discovery) of Spatial-ID demonstrates its promising ability to discover these cell types. (**c**) Inhibitory neurons (Lamp5, Pvalb, Sncg, Sst and Vip).

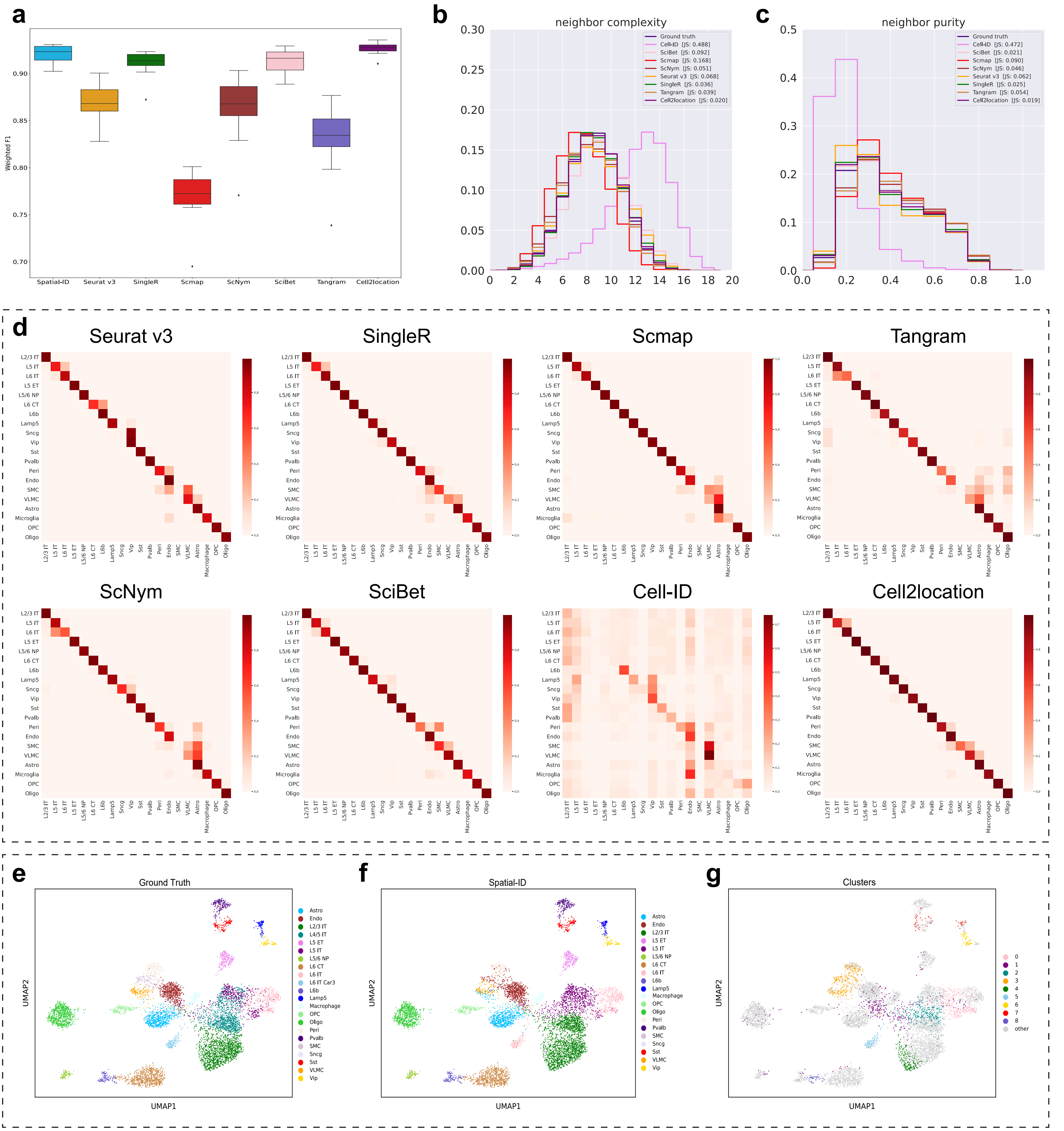

Supplementary Figure 2 | Additional comparisons for the mouse primary motor cortex dataset and UMAP of new cell type discovery. (a) The comparison of mean weighted F1 score; n=12 independent samples; Center line, median; box limits, upper and lower quartiles; whiskers, 1.5× interquartile range. The weighted F1 score of each sample is calculated by weighted averaging the F1 score of each cell type, in order to mitigate the effects of cell type imbalance. Notably, the mean weighted F1 score of Cell-ID is 0.1512, that is far below those shown and is therefore not shown.(**b**) Neighborhood complexity of the control methods. JS: Jensen-Shannon distance. (**c**) Neighborhood purity of the control methods. (**d**) The confusion matrixes of the control methods. (**e**) Visualization of the ground truth of the sample (slice153) using UMAP embedding. (**f**) Visualization of the predictions of Spatial-ID of the sample using UMAP embedding. (**g**) Visualization of the clusters of the unassigned cells using UMAP embedding.

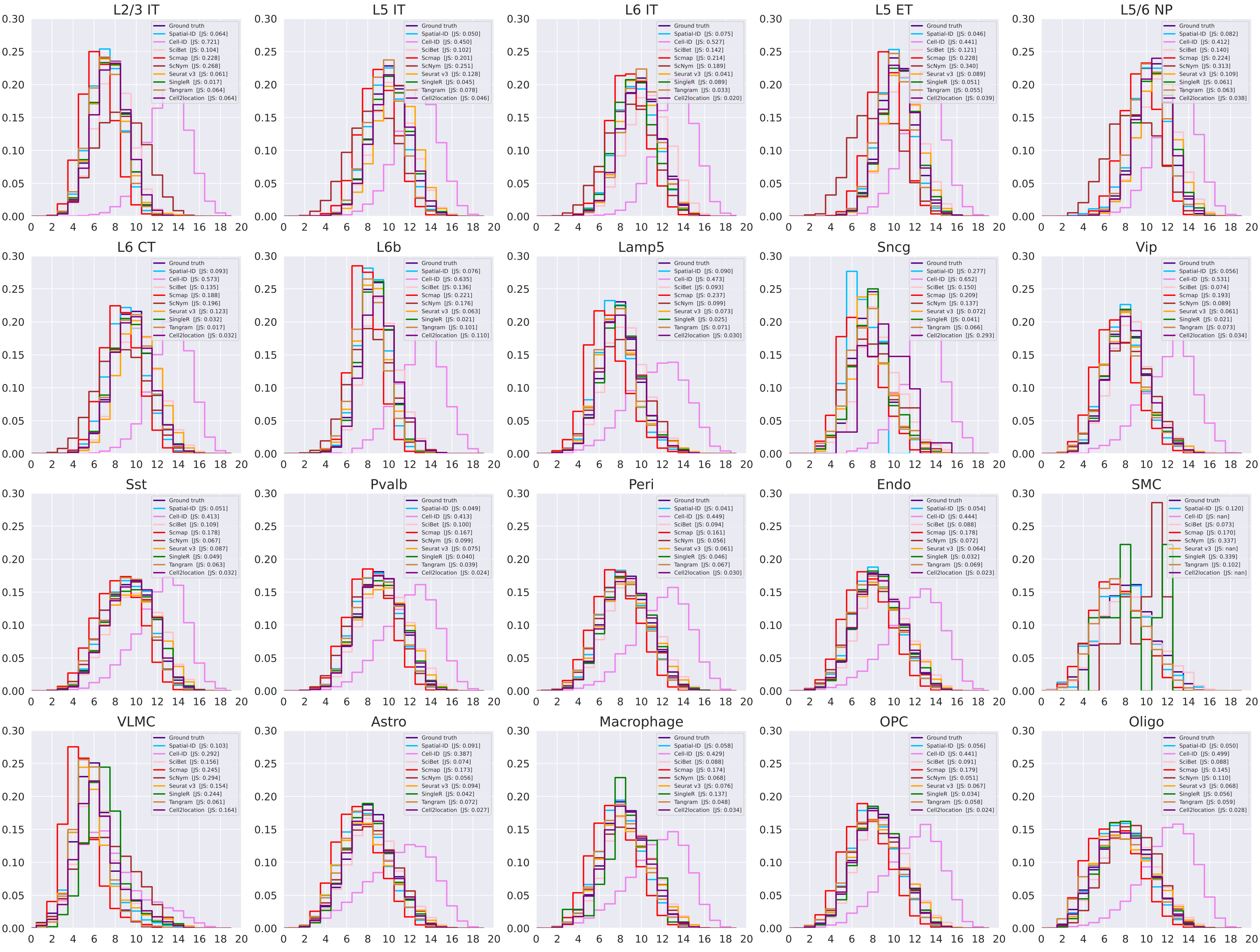

Supplementary Figure 3 | Neighborhood complexity of each cell type in mouse primary motor cortex dataset measured by MERFISH. JS: Jensen-Shannon distance.

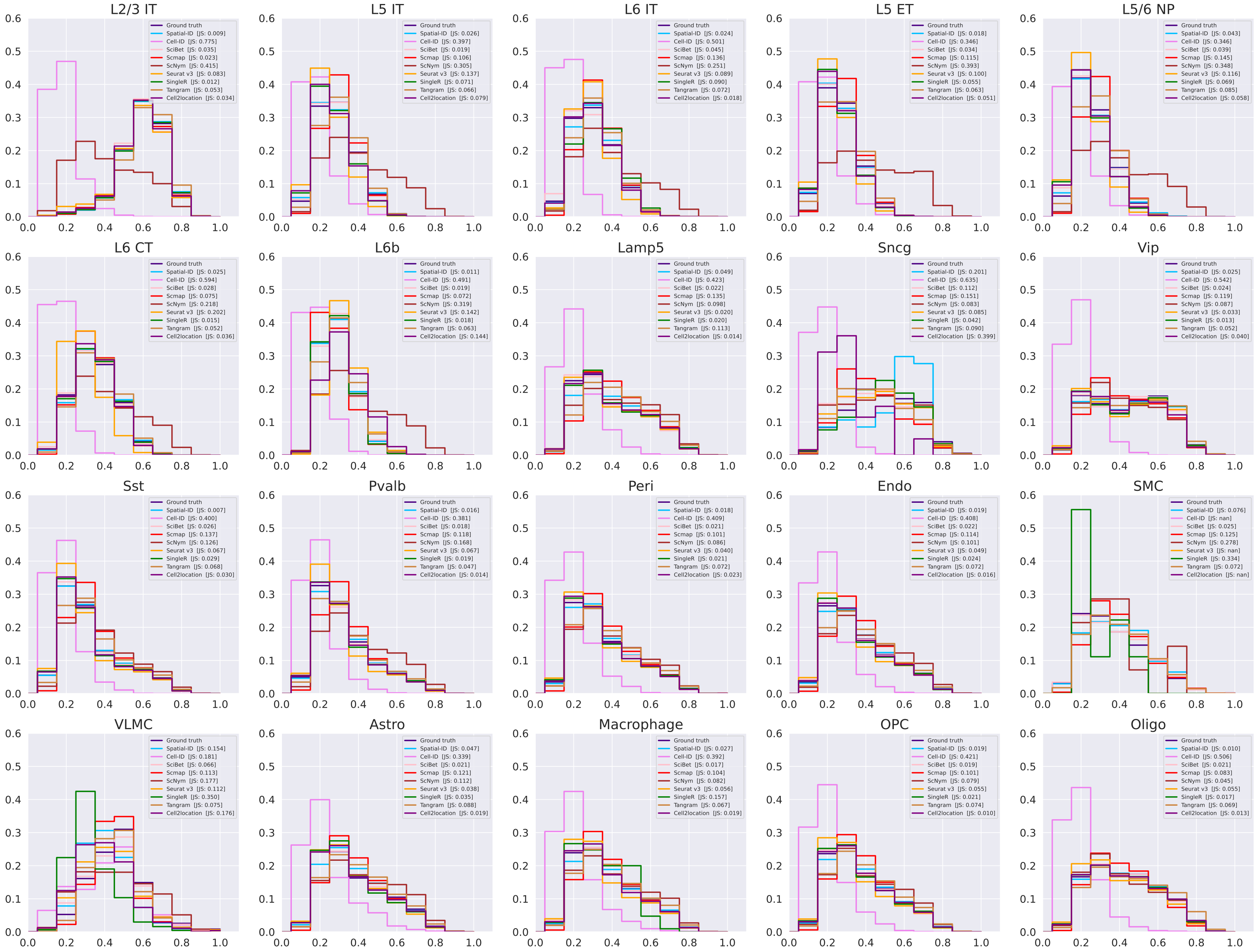

Supplementary Figure 4 | Neighborhood purity of each cell type in mouse primary motor cortex dataset measured by MERFISH. JS: Jensen-Shannon distance.

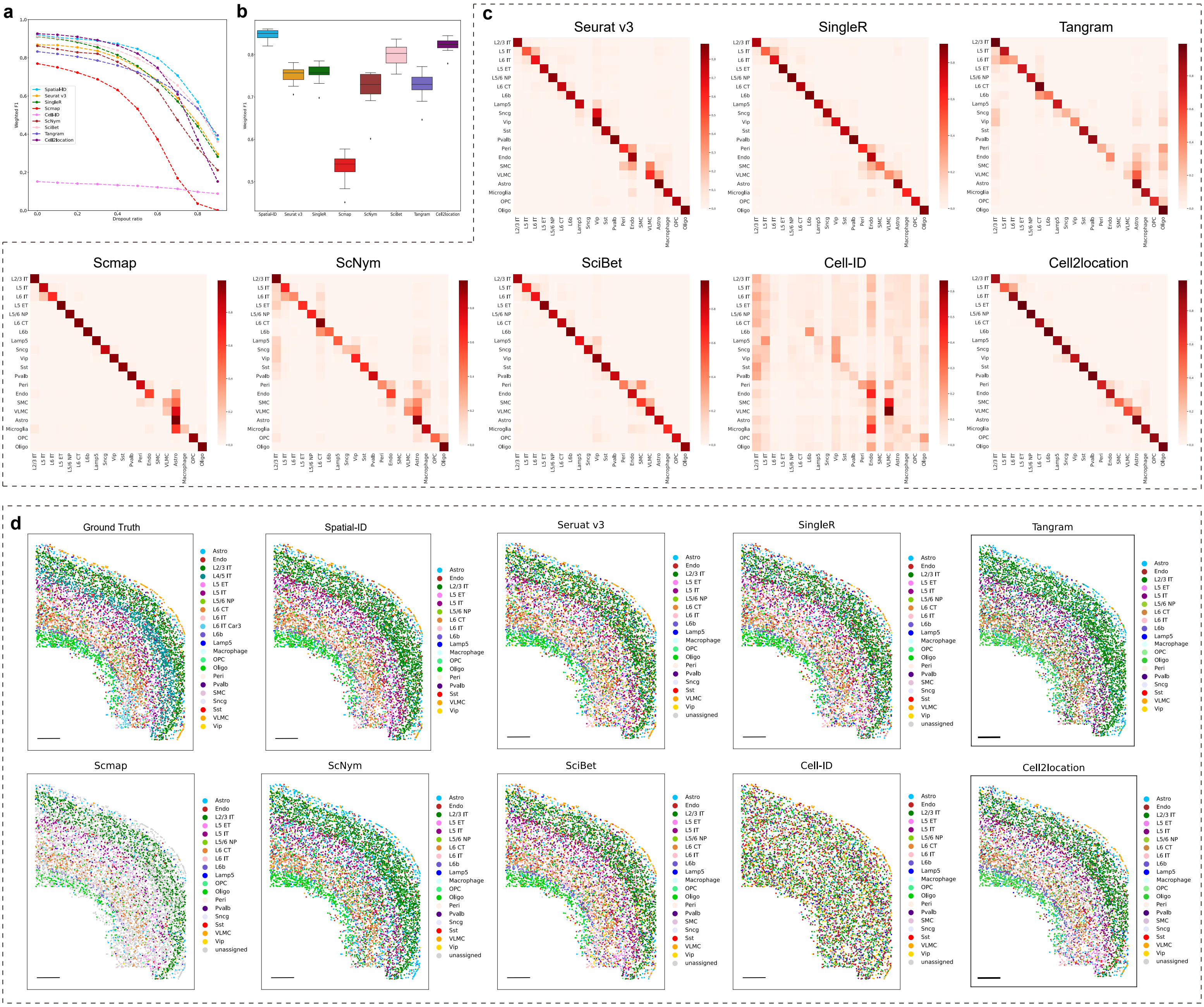

Supplementary Figure 5 | Additional comparisons of different gene dropout rates for the mouse primary motor cortex dataset. (a) The comparison of mean weighted F1 score at different gene dropout rates. **(b)** The comparison of mean weighted F1 score at 0.5 gene dropout rate; n=12 independent samples; Center line, median; box limits, upper and lower quartiles; whiskers, 1.5× interquartile range. Notably, the mean weighted F1 score of Cell-ID is 0.1294, that is far below those shown and is therefore not shown. **(c)** The confusion matrixes of the control methods at 0.5 gene dropout rate. **(d)** Spatial organization of ground truth and predictions of different cell typing methods at 0.5 gene dropout rate**.** Bar scale 400um.

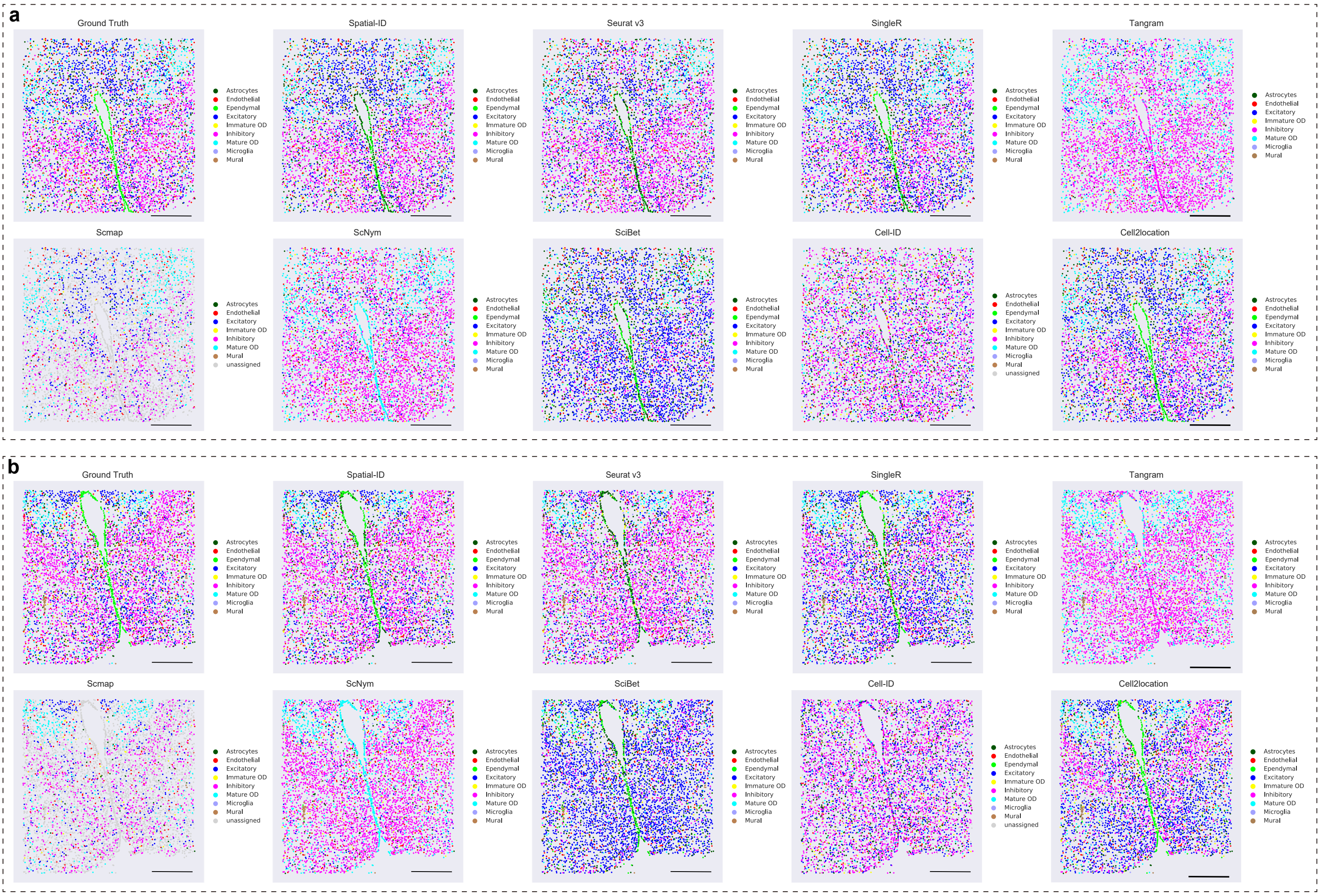

Supplementary Figure 6 | Comparisons of 2D spatial organization for the mouse hypothalamic preoptic region dataset. (a) The slice at Bregma -0.29. Bar scale 400um. **(b)** The slice at Bregma -0.14. Bar scale 400um. Mature OD: Mature oligodendrocyte; Immature OD: Immature oligodendrocyte.

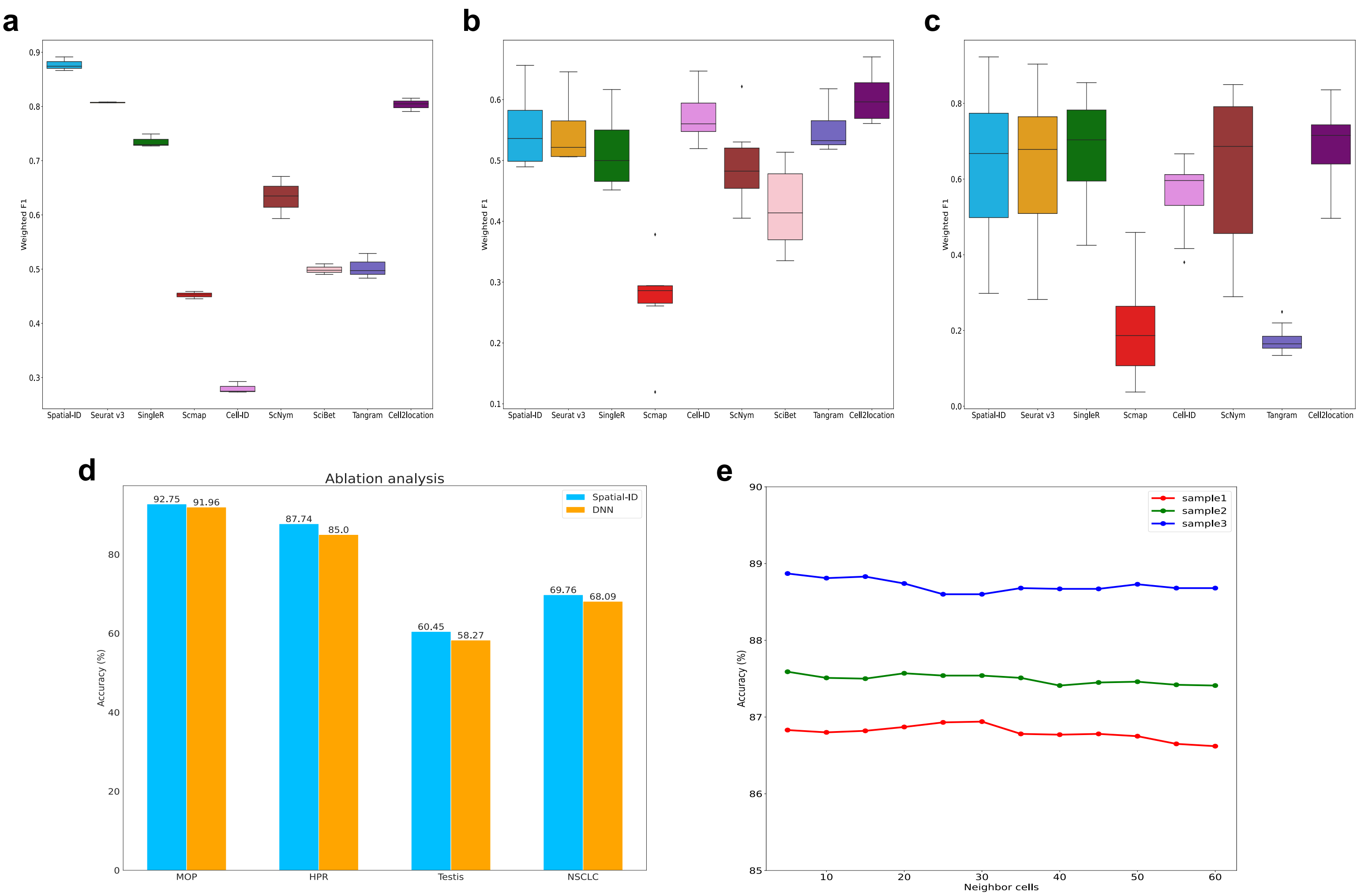

Supplementary Figure 7 | Additional comparisons of mean weighted F1 score. (a) The mouse hypothalamic preoptic region dataset measured by MERFISH; n=3 independent samples; Center line, median; box limits, upper and lower quartiles; whiskers, 1.5× interquartile range. **(b)** The mouse spermatogenesis dataset measured by Slide-seq; n=6 independent samples; Center line, median; box limits, upper and lower quartiles; whiskers, 1.5× interquartile range. **(c)** The human non-small-cell lung cancer (NSCLC) dataset; n=20 independent samples; Center line, median; box limits, upper and lower quartiles; whiskers, 1.5× interquartile range. Notably, the mean weighted F1 score of SciBet is approximately 0, that is far below those shown and is therefore not shown.**(d)** Ablation analysis for Spatial-ID. MOP: mouse primary motor cortex dataset. HPR: mouse hypothalamic preoptic region dataset. Testis: mouse spermatogenesis dataset. **(e)** The effect of spatial information (neighbor cells) on the mouse hypothalamic preoptic region dataset.

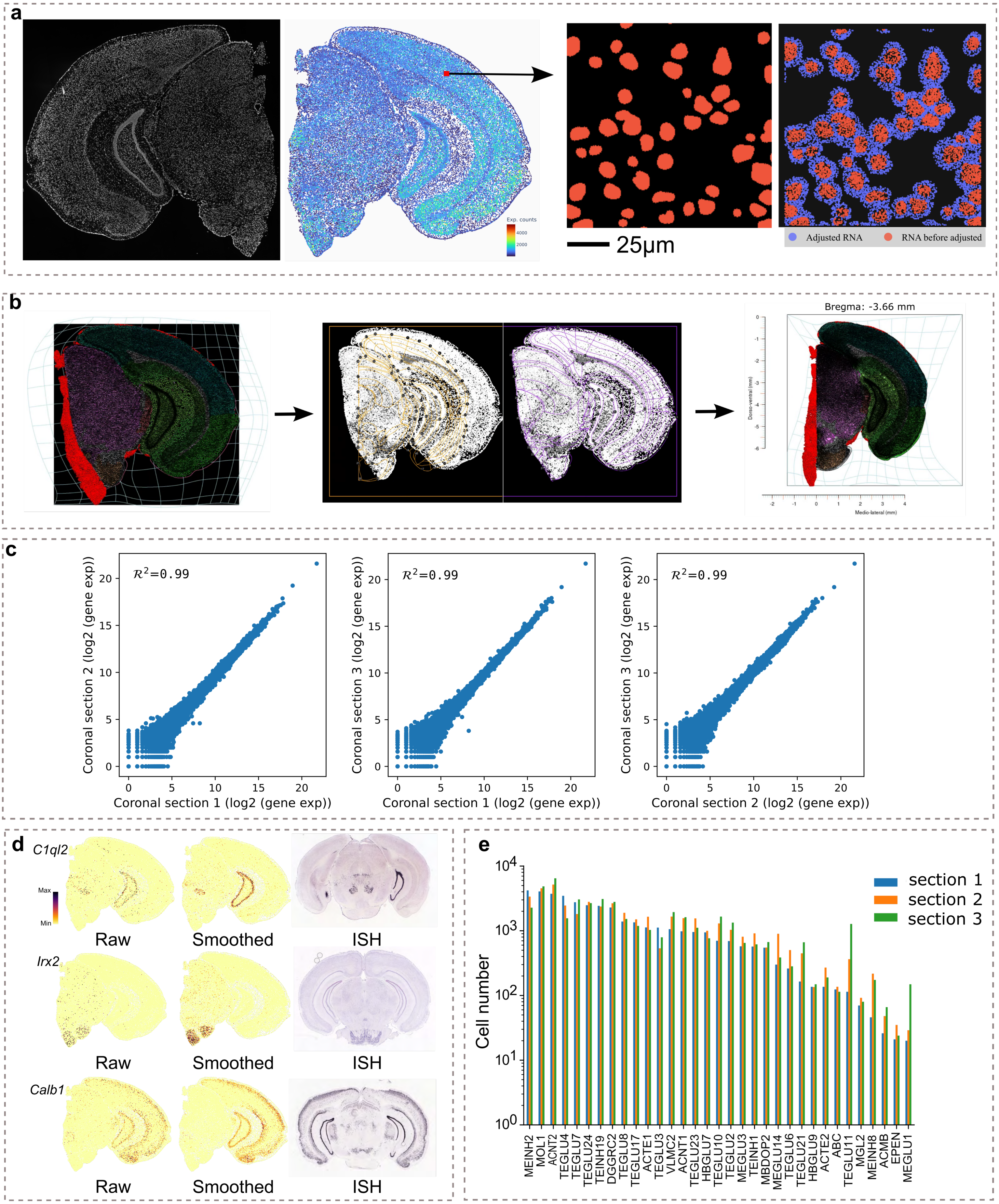

Supplementary Figure 8 | Data processing of the mouse brain hemisphere, and cell number distributions of identified cell types across 3 sections. (**a**) Cell segmentation procedure. ssDNA image of coronal section 3 and the corresponding spatial heatmap that indicates the number of transcripts captured by Stereo-seq are shown in the left panel. The right panel shows the segmented nuclei masks and transcripts surrounding the nucleus adjusted by a Gaussian mixture model. (**b**) The registration procedure is used to align coronal sections with the 3D Allen reference atlas. (**c**) Pearson's correlation coefficients of spatial single-cell profiles between 3 adjacent coronal sections. (**d**) Comparisons between raw expression (left), smoothed by the Gaussian smoothing strategy (middle) and Allen ISH image (right) of selected genes Clql2, Irx2 and Calb1. After Gaussian smoothing, these gene expressions show better spatial distributions that are much closer to their spatial captures of the in situ hybridization of Allen brain atlas. (**e**) Cell number of each identified cell type for the coronal sections 1, 2, and 3, respectively.

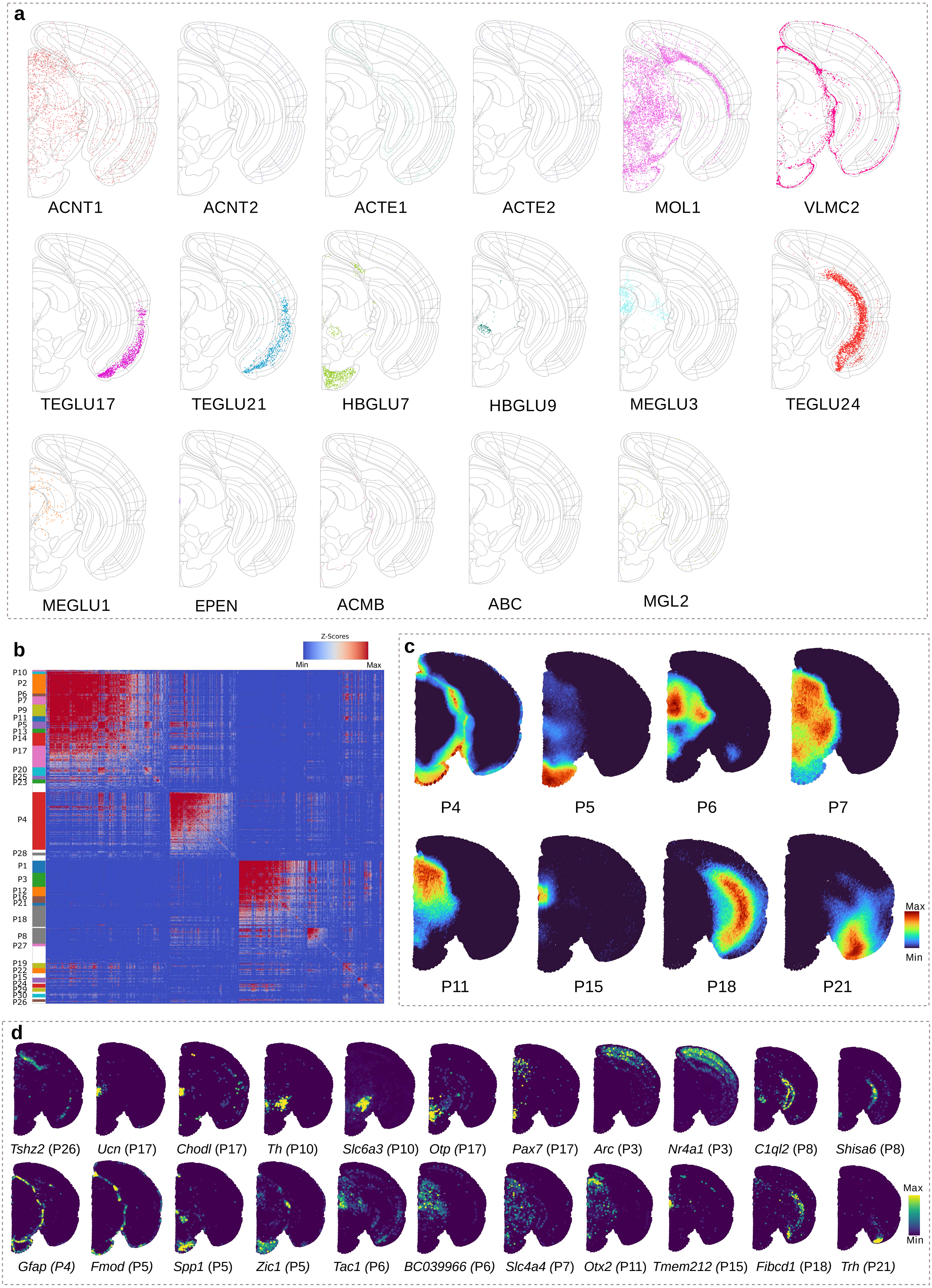
a
ACNT1
ACNT2
ACTE1
ACTE2
MOL1
VLMC2
TEGLU17
TEGLU21
HBGLU7
HBGLU9
MEGLU3
TEGLU24
MEGLU1
EPEN
ACMB
ABC
MGL2
b
Z-Scores
Min
Max
P10
P2
P6
P7
P9
P11
P5
P13
P14
P17
P20
P25
P23
P4
P28
P1
P3
P12
P16
P21
P18
P8
P27
P19
P22
P15
P24
P29
P30
P26
c
P4
P5
P6
P7
P11
P15
P18
P21
Max
Min
d
Tshz2 (P26)
Ucn (P17)
Chodl (P17)
Th (P10)
Slc6a3 (P10)
Otp (P17)
Pax7 (P17)
Arc (P3)
Nr4a1 (P3)
C1ql2 (P8)
Shisa6 (P8)
Gfap (P4)
Fmod (P5)
Spp1 (P5)
Zic1 (P5)
Tac1 (P6)
BC039966 (P6)
Slc4a4 (P7)
Otx2 (P11)
Tmem212 (P15)
Fibcd1 (P18)
Trh (P21)
Max
Min

Supplementary Figure 9 | Additional spatial gene patterns and spatial heatmap of top scored genes. (**a**) Other identified cell types of Section 3. (**b**) Clustered spatial gene patterns. Genes with significant spatial correlation (FDR < 0.05) are clustered into 30 gene patterns on the basis of pairwise spatial correlation. (**c**) Some spatial gene patterns visualize with pattern scores. (**d**) Spatial heatmaps of top scored genes for corresponding spatial gene patterns indicate the number of transcripts captured by Stereo-seq.

Supplementary Table 1 Datasets used in this study.

SRT datasets/ Tissue sample	Techniques	Characteristics						Reference scRNA-seq datasets		
		Samples	Cells	Genes	Field size	Notes	Source of labels	Name	meta information	Source of labels
Mouse brain - primary motor cortex (MOP), (https://doi.brainimagelibrary.org/doi/10.35077/g.21)	High-plex RNA imaging - Multiplexed error-robust FISH (MERFISH)	12	280,186	254	~ 2.0mm× 2.4mm	Samples are collected from 2 mouse brains, where each mouse contains 6 samples, and each sample includes 4-6 coronal slices (10um thick) that are imaged together on the same coverslip.	Cluster analysis and manual labelling	snRNA-seq 10x v3 B, (https://assets.nemoarchive.org/dat-ch1nqb7)	159,738 cells, 31,053 genes.	Cluster analysis and manual labelling
Mouse brain - hypothalamic preoptic region (3D), (https://datadryad.org/stash/dataset/doi:10.5061/dryad.8t8s248)	High-plex RNA imaging - Multiplexed error-robust FISH (MERFISH)	3	213,192	155	1.8mm×1.8mm ×0.6mm	3 samples with naive behavior from 2 female mice and 1 male mouse brains (Bregma 0.26 to -0.29), each sample includes 12 slices with 50um interval.	Cluster analysis and manual labelling	GSE113576, (https://www.ncbi.nlm.nih.gov/geo/query/acc.cgi?acc=GSE113576)	31,299 cells, 27,998 genes.	Cluster analysis and manual labelling
Mouse spermatogenesis, (https://www.dropbox.com/s/ygzpj0d0oh67br0/Testis_Slideseq_Data.zip?dl=0.)	In situ capture, Spatial barcoding - Slide-seq, 10um diameter per sequencing spot.	6	207,335	27,181	~ 2.5mm× 2.5mm	3 samples from leptin-deficient diabetic mice (ob/ob) and 3 samples from wild-type (WT) mice.	non-negative matrix factorization regression (NMF)	GSE112393, (https://www.ncbi.nlm.nih.gov/geo/query/acc.cgi?acc=GSE112393)	34,633 cells, 37,241 genes.	Based on marker genes manually
Human NSCLC - non-small-cell lung cancer (https://nanostring.com/resources/smi-ffpe-dataset-lung9-rep1-data/)	High-plex spatial molecular imaging - CosMx SMI platform (www.nanostring.com), 0.18um per pixel.	20	83,621	980	~ 0.7mm× 0.9mm	20 samples from the individual dataset Lung 9-1 of a 60+ years old patient (section thickness 20um).	Deconvolution-based method and cluster analysis	scRNA-seq NSCLC (https://gbiomed.kuleuven.be/english/research/50000622/laboratories/54213024/scRNAseq-NSCLC)	49,532 cells, 22,180 genes.	Cluster analysis and manual labelling
Mouse brain hemisphere (3D)	In situ capture, Spatial barcoding - Spatio-Temporal Enhanced REsolution Omics-sequencing (Stereo-seq), 0.22um diameter per sequencing spot and 0.5um center-to-center distance.	3	177,077	26,219	~1cm×1cm× 0.02mm	3 adjacent coronal sections (10μm thick, without intervals) along the anterior-posterior axis (Bregma -3.56 to -3.66) from a 5-week-old C57BL/6J male mouse brain.	None	Mouse brain atlas of cell types from the Linnarsson Lab, (http://mousebrain.org/adolescent/)	160,796 cells, 27,998 genes.	Cluster analysis and manual labelling

Supplementary Table 2 The control methods. The robustness indicates the variations of performance across different datasets. The usability indicates the degree of easy-to-use. The efficiency indicates the running efficiency.

Algorithm	Paradigm	Robustness	Usability	Efficiency	GPU	Languages	Mechanism notes	Strategies
SingleR	Correlation-based	✓	✓	-	-	R	SingleR correlates single-cell transcriptomes with reference transcriptomic datasets and improve its inferences by reducing the reference datasets to only top cell types iteratively.	1. Identifying variable genes among cell types in the reference dataset.<br>2. Correlating each single-cell transcriptome with each sample in the reference dataset using Spearman coefficient.<br>3. Reducing the reference dataset to only top cell types by iterative fine-tuning.
Scmap	Correlation-based	-	-	-	-	R	Scmap projects a newly sequenced cell onto a reference dataset by searching the most similar clusters or cells (i.e., nearest neighbors) in the reference dataset and then assigning a cell type if its nearest neighbors have the same cell type.	1. A method conceptually similar to M3Drop is used to select imformative genes, which can overcome batch effects.<br>2. Approximate nearest neighbor search using product quantizer.<br>3. The similarity value are measured by Pearson, Spearman and cosine.
Cell-ID	Correlation-based	-	✓	-	-	R	Cell-ID extracts per-cell gene signatures for a newly sequencing dataset and reference datasets through multiple correspondence analysis (MCA), then uses per-cell gene signatures to perform automatic cell type and functional annotation for target single-cell transcriptomic dataset by cell matching and label transferring from reference datasets.	1. A statistical technique (i.e., MCA) provides a simultaneous representation of cells and genes in low-dimensional space.<br>2. A gene ranking is obtained for each cell in a dataset based on their distance in the MCA space, where the top-ranked genes for a given cell define its gene signature.
ScNym	Supervision-based	✓	-	✓	✓	Python	ScNym employs an adversarial neural network to transfer cell identity annotations from a labeled reference dataset to an unlabeled newly sequencing dataset despite biological and technical differences.	1. A semisupervised adversarial neural network is employed to eliminate domain shifts across different datasets.<br>2. Training and target cell profiles and their labels are augmented using weighted averages.
SciBet	Supervision-based	-	✓	✓	-	R	SciBet uses the mean expression of cell type-specific genes to train a multinomial-distribution model, then calculated the likelihood function of a test cell using the trained model and annotated cell type for the test cell with maximum likelihood estimation.	1. The cell type-specific genes are selected from the training set by E-test.<br>2. The cell type is assigned by the highest likelihood achieved by the model.
Seruat v3	Integration-based	✓	✓	-	-	R	Seurat v3 provides a comprehensive integration strategy to map scRNA-seq data and SRT data, and projected cellular states (e.g., cell type) from a reference dataset to newly generated datasets.	1. Integrating diverse single-cell datasets across technologies and modalities.<br>2. Projecting the datasets into a subspace defined by shared correlation structure across datasets.<br>3. Using canonical correlation analysis (CCA) and mutual nearest neighbors (MNNs) to identify shared subpopulations across datasets.
Tangram	Integration-based	-	✓	-	✓	Python	Tangram employs a deep learning framework based on nonconvex optimization to align sc/snRNA-seq data to various forms of spatial data collected from the same region, then map the spatial distribution of cell types defined by snRNA-seq on spatial slide.	1. The first step, Tangram randomly places the sc/snRNA-seq profiles in space, then computes an objective function that mimics the spatial correlation between each gene in the sc/snRNA-seq data and in the spatial data.<br>2. Then, Tangram rearranges the sc/snRNA-seq profiles in space to maximize the total spatial correlation across the genes shared by the datasets.<br>3. From the learned mapping function, Tangram can map the location of cells of different types.
Cell2locatio n	Integration-based	✓	✓	-	✓	Python	Cell2location employs an interpretable hierarchical Bayesian model to map the spatial distribution of cell types by integrating single-cell RNAseq (scRNA-seq) and multi-cell spatial transcriptomic data from a given tissue.	1.The first step of Cell2location is to estimate reference cell type signatures from scRNA-seq profiles based on Negative Binomial regression.<br>2. The second step, Cell2location decomposes mRNA counts in spatial transcriptomic data using these reference signatures, thereby estimating the relative and absolute abundance of each cell type at each spatial location.

Supplementary Table 3 The average time cost (mins) per sample of Spatial-ID and control methods. Notably, the supervision-based methods (i.e., ScNym and SciBet) do not count the time of models training. For the self-collected mouse brain hemisphere, the average time cost per sample of the control methods are compared in the configuration of 747 marker genes, because correlation-based methods require huge computational resources for tens of thousands of genes.

SRT datasets	average cells per sample, genes	Spatial-ID	Seruat v3	SingleR	Scmap	Cell-ID	ScNym	SciBet	Tangram	Cell2location	Used Cells in Reference datasets
Primary motor cortex (MOP)	22,746 cells, 254 genes	2.4	34.1	50.3	12.5	75.9	0.2	0.3	34.9	50.6	159,738 cells
Hypothalamic preoptic region (3D)	71,064 cells, 155 genes	9.1	11.7	51.3	19.0	32.7	0.1	0.2	19.4	70.9	31,299 cells
Mouse spermatogenesis	34,556 cells, 24015 genes	5.0	11.7	224.0	25.3	39.2	0.4	2.5	29.1	127.8	34,633 cells
Human NSCLC	4,181 cells, 980 genes	0.7	3.9	11.7	1.8	2.8	< 0.1	< 0.1	3.0	15.5	49,532 cells
Mouse brain hemisphere (3D)	59,025 cells, 747 genes	6.1	31.8	285.7	66.0	84.4	0.1	0.2	47.8	169.6	113,488 cells

Supplementary Table 4 The parameters of DNN, autoencoder, variational graph autoencoder and other hyperparameters. The parameters of data preprocess are used for the mouse brain hemisphere dataset. The other SRT datasets do not filter cells and genes with these parameters.

	Parameters	Value	Explanation
Data preprocess	*cell_min_counts*	300 (mouse brain hemisphere dataset)	Cells with total counts fewer than cell_min_counts will be removed.
	cell_max_counts_pct	98% (mouse brain hemisphere dataset)	Cells with total counts higher than cell_max_counts_pct quantile will be removed.
	filter_mt	10% (mouse brain hemisphere dataset)	Cells with percentage of mitochondrial genes larger than 10% will be removed.
	gene_min_cells	10 (mouse brain hemisphere dataset)	Genes presented in fewer than 10 cells will be removed.
DNN	*Fully connected layer*	4	Each fully connect layer is followed by a GRLU layer and dropout layer. Neurons in a fully connected layer have connections to all neurons in the previous layer. 4 fully connect layers contain 128, 64, 64, cell_types neurons for the mouse primary motor cortex dataset, mouse hypothalamic preoptic region dataset and human NSCLC dataset. 4 fully connect layers contain 512, 256, 256, cell_types neurons for the mouse spermatogenesis dataset and mouse brain hemisphere dataset.
	GELU layer	4	A type of activation function layer.
	Dropout layer	4	At training stage, individual neurons are either "dropped out" of the network (ignored) with probability p. Dropout layer is used to reduce overfitting.
	Loss function	Focal Loss	A modified cross entropy loss function is used to alleviate the class imbalance problem.
Autoencoder	*pca_dim*	None	PCA dimension. For the mouse spermatogenesis dataset and the mouse brain hemisphere dataset, this parameter is set as 200. Other datasets use all the shared genes.
	feat_dim	64	The encoded feature dimension.
	Fully connected layer	2	2 fully connect layers contain 100, 64 neurons, respecitively. Each fully connect layer is followed by a batch normal layer, a ELU layer and a dropout layer.
	Batch normal layer	2	Batch normal layer is used to make training of neural networks faster and more stable through normalization of the layers' inputs by re-centering and re-scaling.
	ELU layer	2	A type of activation function layer.
	Dropout layer	2	As above of dropout layer.
	Decoder layer	1	The docoder layer is also a fully connected layer. Neurons in this layers equal to the input dimension (e.g., pca_dim or number of shared genes).
	Loss function	MSE	Mean squared error
Variational graph autoencoder	*sparse GCN layer*	2	A GCN layer receives feature vector of each node and neighbor graph (adjacency matrix). GCN layers contrains 32, 8 neurons, respectively. Each GCN layer is followed by a ReLU layer and a dropout layer.
	RELU layer	2	A type of activation function layer.
	Dropout layer	2	As above of dropout layer.
	Inner-product layer	1	Inner-product layer is used to reconstruct the adjacency matrix by inner product the input tensor.
	Loss function	cross-entropy	Cross entropy loss function
Hyperparameters of Spatial-ID	*edge_weight*	TRUE	Whether to use edge weight in spatial neighbor graph. Using edge weight means nearer neighbors will contribute more to results.
	k_graph	30	Number of neighbors in spatial neighbor graph.
	theta	15	Decay coefficient for edge weight
	w_dae	1	Weight of autoencoder loss.
	w_gae	1	Weight of variantional graph autoencoder loss.
	w_cls	10	Weight of the classifier loss for self-supervised learning.
	epochs	200	Number of training epochs.
	kd_T	1	Temperature parameter for pseudo-labels generated.
New cell type discovery	*Threshold*	0.5~0.9	The threshod in the thresholding step to determine unassigned cells. For the mouse primary motor cortex dataset, the threshold is set as 0.9. For the human NSCLC dataset, the threshold is set as 0.7.
	Clusters	approxinate half of total cell types	The unassigned cells are grouped in to the number of clusters in the clustering step.

Supplementary Table 5 Interpretation of professional terms.

Terms	Explanation
Transfer learning	The definition of transfer learning is that a machine learning model gains problem-solving knowledge from the source domain and stores the knowledge, then the model is applied to solve similar problems in the target domain.
Self-supervised learning	Self-supervised learning is a machine learning paradigm. It contains two steps. The first step generates the pseudo-labels. The second step, the actual task is performed with supervised learning using the pseudo-labels.
Temperature setting strategy	Temperature setting strategy adjusts the parameter called temperature in a standard softmax, then logit values of softmax are converted to pseudo-probabilities (i.e., pseudo-labels in our study), and higher values of temperature have the effect of generating a softer distribution of pseudo-probabilities among the output classes.
Pseudo-label	In contrast to the real labels or ground truth labels, the pseudo-labels are generated by an algorithm.
Fully connected layer	Fully connected layers connect every neuron in one layer to every neuron in another layer
Spatial embedding	Spatial embedding is one of feature learning techniques that allow complex spatial data to be used in neural networks and have been shown to improve performance in spatial analysis tasks.
Spatial neighbor graph	The spatial neighbor graph is a undirected graph defined for a set of points in a metric space, such as the Euclidean space.
Non-negative matrix factorization.	A method commonly used in bioinformatics for dimensionality reduction of gene expression data as the non-negativity constraint reflects that genes are either expressed or not and cannot be negatively expressed.
Probability distribution	In this study, a probability distribution is a mathematical description of the probabilities of cell types for a cell.
Euclidean distance	The Euclidean distance between two points in Euclidean space is the length of a line segment between the two points. It can be calculated from the coordinates of the points using the square root.
Autoencoder	An autoencoder is a type of artificial neural network used to learn efficient codings of input data, which is learned by attempting to regenerate the input from the encoded features.
GCN	A Graph convolutional network (GCN) is a class of neural networks for processing data that can be represented as graphs.
Variational graph autoencoder	Like a variantonal autoencoder, the input data of Variational graph autoencoder is sampled from a parametrized distribution, and the encoder and decoder are trained jointly such that the output minimizes a reconstruction error in the sense of the Kullback–Leibler divergence between the true posterior and its parametric approximation.

Stereo-seq technique process.

Mice were housed under standard laboratory conditions (12 h light/12 h dark cycle, temperature of 21-27 ℃, and humidity of 55-60%) with ad libitum access to water and mouse chow. Mouse brain was collected from a 5-week-old C57BL/6J male mouse. After collection, it was snap-frozen in liquid nitrogen prechilled isopentane in Tissue-Tek OCT (Sakura, 4853), and then transferred to a -80 °C freezer until cryosection. Three adjacent coronal sections (10μm thick, without intervals) along the anterior-posterior axis (Bregma -3.56 to -3.66) were cut from the brain in a -20°C cryostat (Leika, CM1950). Tissue sections were further adhered to the DNA nanoball (DNB) patterned silicon chip surface (Stereo-seq chip, 1 × 1 cm2 in size), and fixed in formaldehyde solution (Sigma, 34860-1L-R) at -20°C for 30 minutes. Each DNB contains a 25 bp randomly barcoded sequence as coordinate identity (CID) for its unique spatial location, having a size of 220 nm in diameter and 500 nm center-to-center distance. After sequencing, 10 bp molecular identifiers (MID) and ployT sequence-containing oligonucleotides were hybridized and ligated to the DNBs. This generated capture probes on the chip containing a 25 bp CID barcode, a 10 bp MID and a ployT ready for in situ RNA capture. Cryosectioned tissues were first stained for 5 minutes with ssDNA Reagent (Thermo fisher, Q10212) for ssDNA visualization. ssDNA images were performed with a Motic PA53 Scanner. Next, tissues were immediately permeabilized by 0.1% pepsin (Sigma, P7000) in 0.01 M HCl (Sigma ,2104-50 mL) buffer (pH = 2) for 12 minutes at 37℃, and followed by in situ reverse transcription using a reverse transcription mix (STOmics, 111ST114)

containing SuperScript II at 42℃ for 3 hours to get the stable cDNAs attached on the chip. Tissues were removed by incubation with Tissue Removal buffer (STOmics, 111ST114) at 55℃ for 10 minutes, which left cDNAs coupled to the patterned DNBs on the chip. We collected the cDNAs by probe cleavage using cDNA Release buffer (STOmics, 111ST114) incubated at 55℃ for 3 hours, and purified them by the Ampure XP Beads (Vazyme, N411-03). The resulting cDNAs were then amplified by polymerase chain reaction (PCR) with cDNA amplification mix (STOmics, 111ST114) containing cDNA primer. PCR reactions were conducted as: first incubation at 95°C for 5 minutes, 15 cycles at 98°C for 20 seconds, 58°C for 20 seconds, 72°C for 3 minutes, and a final incubation at 72°C for 5 minutes. The concentrations of resulting PCR products were quantified by Qubit™ dsDNA Assay Kit (Thermo, Q32854). A total of 20 ng of DNA were fragmented at 55℃ for 10 minutes using Fragment Mix (STOmics, 111ST114), after which the reactions were stopped by the addition of 0.02% SDS buffer and gently mixing at 37°C for 5 minutes after fragmentation. The final fragment products were amplified as described below: 25 μL of fragmentation product, 50 μL PCR Amplification Mix, 25 μL PCR Barcode Primer Mix (STOmics, 111ST114). The reaction was then run as: 1 cycle of 95℃ 5 minutes, 13 cycles of (98℃ 20 seconds, 58℃ 20 seconds and 72℃ 30 seconds) and 1 cycle 72℃ 5 minutes. Library PCR products were purified using the Ampure XP Beads (0.6x and 0.15x), used for DNB generation, and finally sequenced (paired-end 50 bp) on a MGI DNBSEQ-Tx sequencer.
