# Supplementary material for: Spatial-ID: a cell typing method for spatially resolved transcriptomics via transfer learning and spatial embedding
Source: Nat Commun. 2022 Dec 10;13:7640. doi: 10.1038/s41467-022-35288-0 (PMC9741613; doi:10.1038/s41467-022-35288-0)
Supplement: Supplementary file 3 — Reporting Summary [file 41467_2022_35288_MOESM3_ESM.pdf]

nature portfolio

Corresponding author(s): Yuxiang Li, Xun Xu, Jianhua Yao

Last updated by author(s): Nov 21, 2022

Reporting Summary

Nature Portfolio wishes to improve the reproducibility of the work that we publish. This form provides structure for consistency and transparency in reporting. For further information on Nature Portfolio policies, see our Editorial Policies and the Editorial Policy Checklist.

Statistics

For all statistical analyses, confirm that the following items are present in the figure legend, table legend, main text, or Methods section.

n/a	Confirmed	
☐	☒	The exact sample size (n) for each experimental group/condition, given as a discrete number and unit of measurement
☐	☒	A statement on whether measurements were taken from distinct samples or whether the same sample was measured repeatedly
☐	☒	The statistical test(s) used AND whether they are one- or two-sided<br>*Only common tests should be described solely by name; describe more complex techniques in the Methods section.*
☒	☐	A description of all covariates tested
☐	☒	A description of any assumptions or corrections, such as tests of normality and adjustment for multiple comparisons
☐	☒	A full description of the statistical parameters including central tendency (e.g. means) or other basic estimates (e.g. regression coefficient) AND variation (e.g. standard deviation) or associated estimates of uncertainty (e.g. confidence intervals)
☐	☒	For null hypothesis testing, the test statistic (e.g. F, t, r) with confidence intervals, effect sizes, degrees of freedom and P value noted<br>*Give P values as exact values whenever suitable.*
☒	☐	For Bayesian analysis, information on the choice of priors and Markov chain Monte Carlo settings
☒	☐	For hierarchical and complex designs, identification of the appropriate level for tests and full reporting of outcomes
☐	☒	Estimates of effect sizes (e.g. Cohen's d, Pearson's r), indicating how they were calculated

Our web collection on statistics for biologists contains articles on many of the points above.

Software and code

Policy information about availability of computer code

Data collection	The Stereo-seq dataset was collected using an MGI DNBSEQ-Tx sequencer and its control computer. ssDNA images were acquired by Motic PA53 Scanner.
Data analysis	Custom software: https://github.com/TencentAILabHealthcare/spatialID<br>Public softwares: The Stereo-seq dataset was processed using STAR(https://github.com/alexdobin/STAR, V2.7.10a), handleBam(https://github.com/BGIResearch/handleBam). Other analysis softwares include Hotspot(https://github.com/Yoseflab/Hotspot, V0.9.1), Scanpy (https://github.com/scverse/scanpy, V1.8.1), Seurat(https://satijalab.org/seurat/, V4.0), wholebrain(https://github.com/tractatus/wholebrain), ScNym(https://github.com/calico/scnym, v0.3), Scmap(http://bioconductor.org/packages/scmap, V1.20.0), SciBet(https://github.com/PaulingLiu/scibet), Cell-ID(https://github.com/RausellLab/CelliD, v1.21), SingleR(https://github.com/dviraran/SingleR, v1.0), numpy==1.21.3, pandas==1.2.4, scipy==1.5.4, matplotlib==3.3.4, seaborn==0.11.1, scikit-learn==0.24.2, torch==1.8.1, torch_geometric==1.7.2

For manuscripts utilizing custom algorithms or software that are central to the research but not yet described in published literature, software must be made available to editors and reviewers. We strongly encourage code deposition in a community repository (e.g. GitHub). See the Nature Portfolio guidelines for submitting code & software for further information.

Data

Policy information about availability of data

All manuscripts must include a data availability statement. This statement should provide the following information, where applicable:

- Accession codes, unique identifiers, or web links for publicly available datasets
- A description of any restrictions on data availability
- For clinical datasets or third party data, please ensure that the statement adheres to our policy

Data availability was described in the manuscript.
The raw Stereo-seq sequencing data used in this study have been deposited to China National Gene Bank (CNGB) Sequence Archive (accession code: CNP0002966, https://db.cngb.org/search/project/CNP0002966/), that are available from the corresponding author upon reasonable request. The raw and processed Stereo-seq sequencing data have been deposited in Zenodo (DOI: 10.5281/zenodo.7340795) that are publicly accessible.
Besides, the public datasets are freely available as follow. Mouse brain - primary motor cortex (MOP): doi/10.35077/g.21 (https://doi.brainimagelibrary.org/doi/10.35077/g.21). Mouse brain - hypothalamic preoptic region: doi:10.5061/dryad.8t8s248 (https://datadryad.org/stash/dataset/doi:10.5061/dryad.8t8s248). Mouse spermatogenesis: Testis_Slideseq_Data.zip (https://www.dropbox.com/s/ygzpj0d0oh67br0/Testis_Slideseq_Data.zip?dl=0). Human NSCLC: SMI-FFPE Dataset–Lung9-Rep1 Data (https://nanostring.com/resources/smi-ffpe-dataset-lung9-rep1-data/). The snRNA-seq 10x v3 B of BICCN MOP dataset (RRID: SCR_015820) can be accessed via the NeMO archive (RRID: SCR_002001) at accession: (https://assets.nemoarchive.org/dat-ch1nqb7). scRNA-seq of preoptic region of mouse hypothalamic: GSE113576 (https://www.ncbi.nlm.nih.gov/geo/query/acc.cgi?acc=GSE113576). scRNA-seq of mouse testis: GSE112393 (https://www.ncbi.nlm.nih.gov/geo/query/acc.cgi?acc=GSE112393). scRNA-seq NSCLC: 54213024/scRNAseq-NSCLC (https://gbiomed.kuleuven.be/english/research/50000622/laboratories/54213024/scRNAseq-NSCLC). Mouse brain atlas of cell types from the Linnarsson Lab: SRP135960 (http://mousebrain.org/adolescent).

Human research participants

Policy information about studies involving human research participants and Sex and Gender in Research.

Reporting on sex and gender	N/A
Population characteristics	N/A
Recruitment	N/A
Ethics oversight	N/A

Note that full information on the approval of the study protocol must also be provided in the manuscript.

Field-specific reporting

Please select the one below that is the best fit for your research. If you are not sure, read the appropriate sections before making your selection.

☒ Life sciences ☐ Behavioural & social sciences ☐ Ecological, evolutionary & environmental sciences

For a reference copy of the document with all sections, see nature.com/documents/nr-reporting-summary-flat.pdf

Life sciences study design

All studies must disclose on these points even when the disclosure is negative.

Sample size	The four public available SRT datasets (i.e., mouse brain primary motor cortex, mouse brain hypothalamic preoptic region, mouse spermatogenesis and human non-small-cell lung cancer) contained 12, 3, 6, 20 samples, respectively. For Stereo-seq dataset, three adjacent sections were imaged from the same animal for the prediction of cell types. In total, ~180000 cells were obtained, which generated a sufficient number of spatial single-cell profiles.
Data exclusions	No data was excluded from the study.
Replication	Analysis results of each dataset were replicated from all the samples in the same dataset.
Randomization	Not applicable. Only one animal was chosen and no randomization was necessary for this study.
Blinding	Not applicable. Blinding was not needed in this study because all sections were collected and sequenced under the same condition. Blinding was not used in this study.

Reporting for specific materials, systems and methods

We require information from authors about some types of materials, experimental systems and methods used in many studies. Here, indicate whether each material, system or method listed is relevant to your study. If you are not sure if a list item applies to your research, read the appropriate section before selecting a response.

Materials & experimental systems

n/a	Involved in the study
☒	☐ Antibodies
☒	☐ Eukaryotic cell lines
☒	☐ Palaeontology and archaeology
☐	☒ Animals and other organisms
☒	☐ Clinical data
☒	☐ Dual use research of concern

Methods

n/a	Involved in the study
☒	☐ ChIP-seq
☒	☐ Flow cytometry
☒	☐ MRI-based neuroimaging

Animals and other research organisms

Policy information about studies involving animals; ARRIVE guidelines recommended for reporting animal research, and Sex and Gender in Research

Laboratory animals	Stereo-seq dataset in this study was collected from a 5-week-old C57BL/6J laboratory mouse. The Mouse was housed under standard laboratory conditions （12 h light/12 h dark cycle, temperature of 21-27℃, and humidity of 55-60%) with ad libitum access to water and mouse chow.
Wild animals	Not applicable. The study did not involve wild animals.
Reporting on sex	Findings of Stereo-seq dataset apply only to male sex of mouse, and sex was not considered in this study design.
Field-collected samples	Not applicable. No field-collected samples were used in this study.
Ethics oversight	All experimental protocols for generating Stereo-seq dataset presented in this study were compliant with ethical regulations regarding animal research and approved by the Animal Care and Use committee of the Guangzhou Institutes of Biomedicine and Health, Chinese Academy of Sciences and the Institutional Review Board of BGI, Shenzhen, China (Ethical permit license number: BGI-IRB A21001).

Note that full information on the approval of the study protocol must also be provided in the manuscript.
